# Seroprevalence of Hepatitis A among Thai Population Residing Near Myanmar Border

**DOI:** 10.3329/jhpn.v29i2.7861

**Published:** 2011-04

**Authors:** Pornpimol Rianthavorn, Apinya Fakthongyoo, Siriwan Yamsut, Apiradee Theamboonlers, Yong Poovorawan

**Affiliations:** ^1^Center of Excellence in Clinical Virology, Department of Pediatrics, Faculty of Medicine, Chulalongkorn University, Bangkok 10330, Thailand; ^2^Umphang Hospital, Tak, Thailand

**Keywords:** Hepatitis A, Seroprevalence, Myanmar, Thailand

## Abstract

When compared with Thailand, the seroprevalence of hepatitis A virus (HAV) is extremely high among its neighbouring countries. To investigate the seroprevalence of HAV among the Thai people residing in the border area between Thailand and Myanmar, 308 residents in Umphang, Maesod district, Tak, were recruited. Sera were tested for HAV IgG antibodies by enzyme-linked immunosorbent assay. The overall seroprevalence among the Thai people residing in the border area of Thailand was significantly higher than that among the general Thai population (71% vs 27% respectively, p<0.05). As asymptomatic or mild HAV infection typically occurs in children, the Thai people residing in the border area may receive little benefit from universal HAV vaccination. Lower protective antibodies against HAV, along with the exclusion of HAV vaccine from the Expanded Programme on Immunization, potentially increase the susceptibility to HAV among the general Thai population and may lead to more future outbreaks if HAV is introduced from the border areas. The findings suggest that HAV vaccines should be recommended to travellers before their journey to the border between Thailand and Myanmar where HAV is endemic.

## INTRODUCTION

The seroprevalence of hepatitis A virus (HAV) in most parts of Asia has gradually declined as a result of significant improvements in hygiene and living standards (1-3). Least-developed countries of the region, including Myanmar, Laos, and Cambodia, have relatively little impact of these improvements. Although the true seroprevalence of HAV in these least-developed countries is yet to be determined, the seroprevalence is expected to be high. This estimation has been derived from the surveillance study of HAV antibodies among immigrant workers from Myanmar, Laos, and Cambodia in Thailand. Immigrant workers from Myanmar had the highest rate (100%) of HAV seroprevalence, and this observation most likely reflects the high prevalence of HAV in Myanmar (4).

**Fig. 1:**
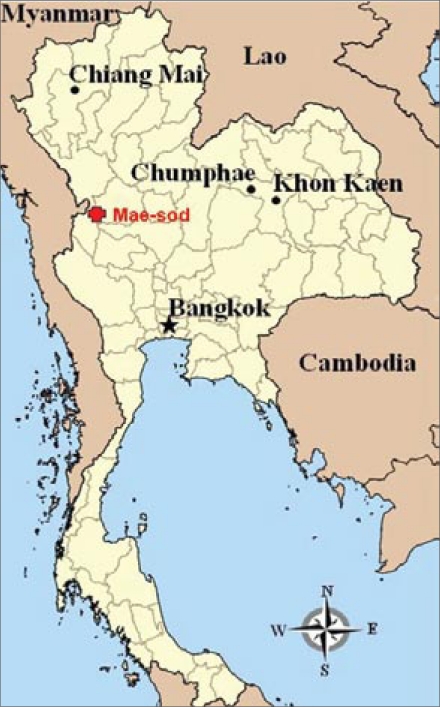
Thailand is represented in yellow whereas its neighbouring countries, including Myanmar, Laos, and Cambodia, are represented in brown on the map. Maesod district in Tak province is in the border area between Thailand and Myanmar and is represented by a red cross

The seroprevalence of HAV among the Thai people residing in the border area adjacent to Myanmar is yet to be studied, although, due to geographical proximity, it is presumed to be higher than that among the general Thai population. The Expanded Programme on Immunization (EPI) in Thailand has excluded HAV vaccine based on cost-effective analysis, indicating that the low incidence of HAV infection, along with the cost of the vaccine, tempers its cost-effectiveness (5). Yet, the EPI for the general Thai population may not be applicable to the Thai people residing in the border area. In this report, we investigated the seroprevalence of HAV infection among residents in Umphang, Maesod district, Tak, Thailand. Tak province is one of the border provinces between Thailand and Myanmar (Fig. 1), and residents of Tak province were selected to represent the Thai people residing in the border area.

## MATERIALS AND METHODS

The participants included either healthy children who had attended the well-child clinic or patients with acute illness who had attended the outpatient clinic or had been hospitalized at the Umphang Hospital, Tak, Thailand, during January 2009–June 2010.

Sera were collected from the subjects and stored at −20 ^o^C until tested for anti-HAV by a commercial ELISA (Architect^®^ HAVAb-IgG; Abbott, Wiesbaden, Germany) at the Center of Excellence in Clinical Virology, Chulalongkorn University, Thailand. The seroprevalence of HAV in each age-group was calculated and reported as percentage. The age-related seroprevalence of HAV among the people residing in the border area was compared with that of Thai (6) and Myanmar immigrant workers (4) previously reported. The differences of proportions of overall HAV seroprevalence in each population were assessed by chi-square test, using the SPSS software (version 13) (SPSS, Chicago, IL). Probability values of less than 5% were considered to be significant.

### Ethical issues

The Institutional Review Board of the Faculty of Medicine, Chulalongkorn University, approved the study. Informed consent was obtained at the time of enrollment. The specimens were used with permission from the Director of the Umphang Hospital, and patient anonymity was maintained. The specimens were used solely for academic purposes.

## RESULTS

Overall, 308 subjects—149 males and 159 females—were recruited. The subjects were divided by age, as follows: five were aged less than one year, 67 were aged 1-6 years, 16 aged 7-12 years, 73 aged 13-20 years, 45 aged 21-30 years, 33 aged 31-40 years, 23 aged 41-50 years, 23 aged 51-60 years, and 23 aged over 60 years. The overall seroprevalence of HAV was 71%. The seroprevalence positively correlated with the age of the subjects, except for the infancy period (less than 1 year old), which had a high seroprevalence of 60%. By the age of 40 years, almost every subject had developed HAV IgG antibodies.

**Fig. 2:**
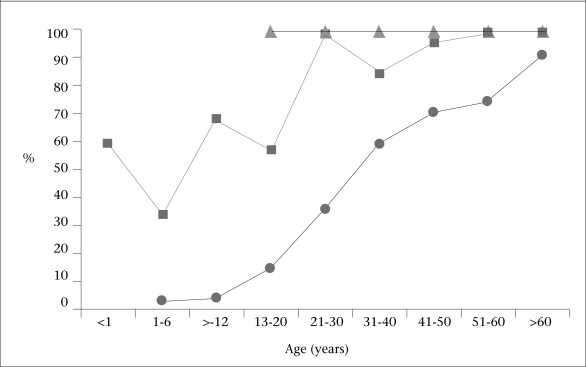
Age-related seroprevalence of hepatitis A virus among Thai population residing near Myanmar border (▪), general Thai population (6) (•), and Myanmar immingrant workers (4) (▴)

When comparing the seroprevalence of HAV among the people residing in the border area with that of Thai (6) and Myanmar (4) populations, the age-related seroprevalence of HAV among the people residing in the border area was significantly higher than that of the general Thai population (p<0.05) but lower than that of Myanmar immigrant workers (p<0.05) (Fig. 2).

## DISCUSSION

HAV remains a major public-health problem wherever hygiene and sanitation are inadequate. Accordingly, the border area of Thailand is prone to sporadic outbreaks of HAV as previously reported in a study by Barameechai *et al*. (7). The seroprevalence of HAV among the general Thai population has declined, especially in children and young adults, during the past two decades while the seroprevalence of HAV in the neighbouring countries has been persistently high.

The age-related anti-HAV seroprevalence among the people residing in the border area between Thailand and Myanmar was in between that observed among the Thai population and Myanmar immigrant workers in previous studies. The seroprevalence in all three populations increased in relation to the age of the subjects and reached its peak with the Thai people residing in the border area who, on average, were older than Myanmar immigrant workers but younger than positive subjects within the general Thai population. As it has been previously shown that HAV IgG antibodies can be trans-placentally transferred (8), the spike of seroprevalence in infancy merely reflected the seroprevalence of HAV infection among women of childbearing age which was almost equivalent to the overall seroprevalence among this population.

The results of the present study reflect the hygiene and sanitation conditions in the border area of Thailand which may have had relatively little improvement when compared with the other parts of the country. It was reported that HAV infection in children usually exhibits mild non-specific symptoms and low case-fatality compared to HAV infection in adults (9, 10). Figure 2 shows that the seroprevalence of HAV was higher among children residing in the border area than among the general Thai population. Thus, the incidence of severe acute hepatitis A might be lower in this area as children infected with HAV during childhood usually experience no or mild symptoms and acquire protection against future symptomatic acute hepatitis A which usually occurs in adults. Therefore, universal HAV vaccination might be even less cost-effective in the Thai population residing in the border area than in the general Thai population to whom universal HAV vaccination is currently not recommended based on the cost-effictive analysis (5). Further studies on cost-effectiveness based on the high seroprevalence of HAV infection among the Thai population residing in the border area are necessary for future EPI recommendations for Thailand. Lower protective antibodies against HAV, along with the exclusion of HAV vaccine from the EPI, potentially increase the susceptibility of the general Thai population to HAV and may lead to more future outbreaks if HAV is introduced from the border areas. HAV vaccines should be recommended to travellers before their journey to the border between Thailand and Myanmar where HAV remains endemic.

## ACKNOWLEDGEMENTS

The authors express their deep gratitude to the Commission on Higher Education, Ministry of Education, Department of Medical Sciences, Ministry of Public Health, and Center of Excellence in Viral Hepatitis, Thailand Research Fund, Center of Excellence in Clinical Virology, Chulalongkorn University, CU Centenary Academic Development Project, and King Chulalongkorn Memorial Hospital for their generous support. Finally, they thank Ms Petra Hirsch for reviewing the manuscript.
